# Brief Research Report: Expression of PD-1 and CTLA-4 in T Lymphocytes and Their Relationship With the Periparturient Period and the Endometrial Cytology of Dairy Cows During the Postpartum Period

**DOI:** 10.3389/fvets.2022.928521

**Published:** 2022-07-22

**Authors:** Carolina Menezes Suassuna de Souza, Ewerton de Souza Lima, Raphael Ferreira Ordonho, Bianca Rafaella Rodrigues dos Santos Oliveira, Rebeca Cordeiro Rodrigues, Marquiliano Farias de Moura, Daniel Magalhães Lima, Maiara Garcia Blagitz, Eduardo Milton Ramos Sanchez, Isac Almeida de Medeiros, Fernando Nogueira Souza, Artur Cezar de Carvalho Fernandes

**Affiliations:** ^1^Programa de Pós-Graduação em Ciência Animal, Centro de Ciências Agrárias, Universidade Federal da Paraíba, Areia, Brazil; ^2^Núcleo Aplicado à Produção e Sanidade da Glândula Mamária (NAPROSA), Departamento de Ciências Veterinárias, Centro de Ciências Agrárias, Universidade Federal da Paraíba, Areia, Brazil; ^3^Departamento de Ciências Veterinárias, Centro de Ciências Agrárias, Universidade Federal da Paraíba, Areia, Brazil; ^4^Departamento de Medicina Veterinária Preventiva e Saúde Animal, Faculdade de Medicina Veterinária e Zootecnia, Universidade de São Paulo, São Paulo, Brazil; ^5^Programa de Pós-Graduação em Saúde, Bem-Estar e Produção Animal Sustentável na Fronteira Sul, Universidade Federal da Fronteira Sul, Realeza, Brazil; ^6^Veterinary Clinical Immunology Research Group, Departamento de Clínica Médica, Faculdade de Medicina Veterinária e Zootecnia, Universidade de S3ão Paulo, São Paulo, Brazil; ^7^Department of Public Health, School of Health Sciences, National University Toribio Rodriguez de Mendoza of Amazonas, Chachapoyas, Peru; ^8^Laboratório de Sorologia e Imunobiologia, Instituto de Medicina Tropical, Universidade de São Paulo, São Paulo, Brazil; ^9^Departamento de Ciências Farmacêuticas, Centro de Ciências da Saúde, Universidade Federal da Paraíba, João Pessoa, Brazil

**Keywords:** immune checkpoints, endometrial cytology, transition period, endometritis, dairy cow

## Abstract

The present study sought to evaluate the expression of PD-1 and CTLA-4 in blood T lymphocytes during the periparturient period and their relationship with uterine health in dairy cows, as determined by endometrial cytology and serum concentrations of β-hydroxybutyrate (BHB) and non-esterified fatty acids (NEFAs), which are indicators of a negative energy balance. The second objective of this study was to investigate whether the expression of PD-1 and CTLA-4 in T lymphocytes is associated with the serum acute phase-protein haptoglobin concentration during the periparturient period. To address these objectives, 26 clinically healthy dairy cows were used. Peripheral blood was collected 14 days prepartum (T-14), at calving (T0), and 30 days postpartum (T30) to measure the expression of PD-1 and CTLA-4 in blood T lymphocytes by flow cytometry. In addition, we collected blood at T0, 10 days after parturition (T10), and T30 to obtain serum and determine the serum concentrations of NEFA, BHB, and Hp. Endometrial cytology was performed at T10, 20 days after parturition (T20), and T30. In the present study, we observed higher expression of CTLA-4 and PD-1 in T lymphocytes at parturition and in the prepartum period, which could indicate a relationship between these immune checkpoints and immunological tolerance during gestation in dairy cattle. In addition, a negative association between the expression of these immune checkpoints prepartum or at parturition and endometrial cytology at T20 and T30 was observed, indicating the negative implications of these immune response regulators in susceptibility to infections. This finding was further corroborated by the relationship between the serum concentration of haptoglobin and the expression of CTLA-4 and PD-1 by T lymphocytes. However, we did not observe a relationship between the indicators of negative energy balance, evaluated by the serum concentrations of BHB and NEFA, and the expression of the immune checkpoint markers studied. Thus, our findings represent an initial step that paves the way for the development of new therapeutic alternatives directed by the host with the objective of increasing the resistance of dairy cattle to infections in this critical period of life.

## Introduction

The transition period in dairy cows between the 3 weeks preceding calving and 3 weeks after calving is characterized by drastic metabolic and immune response changes. During this period, dairy cows are usually in a state of negative energy balance that results in substantial lipid mobilization, a drop in glucose levels, and a dramatic rise in nonesterified fatty acid (NEFA) and β-hydroxybutyrate (BHBA) levels, which impairs immune cell functions (1, 2). Thus, dairy cows face the highest incidence of infectious diseases and metabolic disorders during this period (3, 4). For instance, dairy cows are often affected by endometritis during the postpartum period, which has a substantial negative impact on subsequent reproductive performance (5, 6). This condition is typically detected through endometrial cytology assessment and is referred to as cytological endometritis (6).

Although most dairy cows are subject to bacterial contamination of the uterus during the postpartum period, only a fraction of these animals develop the disease. However, the mechanisms that determine the process by which some animals resolve uterine infection, while others resist it, are not yet fully understood (7). In this context, the investigation of immune checkpoint inhibitors, particularly programmed cell death protein 1 (PD-1) and cytotoxic T lymphocyte-associated antigen-4 (CTLA-4), has gained prominence in recent years, as these molecules have been linked to immunosuppression, disease progression, and a poor prognosis (8, 9), including in bovine infectious diseases (10–12). These molecules, PD1 and CTLA-4, are vital in maintaining self-tolerance and modulating the magnitude and duration of effector immune responses to prevent collateral tissue damage. Signaling by these molecules may induce 'exhaustion' in effector immunological cells (particularly T lymphocytes). T-cell exhaustion is characterized by decreased effector function, persistent expression of immunological checkpoint molecules, poor recall responses, and a transcriptional state that differs from that of functional effector or memory T cells. Thus, there is emerging evidence that several pathogens promote inhibitory interactions between immune cells through immune checkpoint proteins to evade the host immune system by exploiting immunological tolerance and restricting immune-mediated pathogen clearance (8). Investigations into these immunosuppressive interactions have the potential to develop clinical strategies for treating and controlling veterinary infectious diseases by boosting the body's natural immune response to fight diseases, similar to how antibodies that block these immune checkpoints have been used to treat and control many types of malignancies (8, 13).

However, the relationships between these immune checkpoints and the transition period in dairy cows and uterine health in the postpartum period in dairy cows have not yet been studied. Thus, the present study sought to evaluate the expression of PD-1 and CTLA-4 in blood T lymphocytes during the periparturient period and their relationship with uterine health, as determined by endometrial cytology, and serum concentrations of β-hydroxybutyrate (BHB) and non-esterified fatty acids (NEFAs), widely used indicators of negative energy balance.

Furthermore, the measurement of acute-phase proteins is widely used in the diagnosis of diseases in ruminants and is an important indicator of inflammatory processes in cattle (14), in which haptoglobin (Hp) stands out for its high sensitivity and specificity and ability to discriminate between acute and chronic infections (15). Therefore, Hp is the most studied acute-phase protein and is used to detect cows with a high risk of systemic disease and severe postpartum inflammation (16). Hence, the second objective of the present study was to investigate whether the expression of PD-1 and CTLA-4 in blood T lymphocytes is associated with the serum Hp concentration during the periparturient period.

## Materials and Methods

### Animals and Experimental Design

For the present study, 26 clinically healthy dairy cows, including 19 multiparous cows (between their 2^nd^ and 5^th^ lactation; 3.26 ± 0.21) and 7 primiparous cows from two dairy farms, were used. Among these animals, 16 animals of the Guzerá breed (Farm A) and 10 animals of the Girolando breed, composed of the crossbreeding of Holstein and Gir cattle (Farm B), were used. At the first sampling, only healthy cows were enrolled, with no detectable clinical illness. Peripheral blood was collected 14 days prepartum (T-14), at calving (T0), and 30 days postpartum (T30) to measure the expression of PD-1 and CTLA-4 in blood T lymphocytes. In addition, we collected blood at T0, 10 days after parturition (T10), and T30 to obtain serum and determine the serum concentrations of NEFA, BHB, and the acute-phase protein haptoglobin. Endometrial cytology was performed at T10, 20, days after parturition (T20) and T30.

In herd A, the Guzerá cows were kept on Massai grass (a spontaneous hybrid between *Panicum maximum* and *Panicum infestum*) pasture and received soybean meal, corn meal, and cottonseed meal and cake as the concentrates according to their milk production, as well as vitamins and mineral supplements. These zebu dairy cows were milked once a day by manual milking with the calf at foot and yielded an average of 20 kg milk/day, and the calves were maintained half of the day with their dams, as the high weight of the calves was prioritized due their high economic value. Both artificial insemination and natural mating were used in this herd. In herd B, the dairy cows were maintained part of the time on Mombaça grass (*Panicum* maximum) pasture (in the morning) and received palm as the roughage and soybean meal, corn meal, and cottonseed meal as the concentrates according to their milk production, as well as vitamins and mineral supplements. These dairy cows were milked twice daily using machine milking and yielded an average of 27 kg milk/day, and all cows were artificially inseminated in this herd.

### Collection of Peripheral Blood Samples

The peripheral blood samples were collected aseptically by venipuncture of the jugular vein in vacutainer® tubes containing sodium heparin (T-14, T0, and T30; two tubes with 4 mL per sampling time; cat. no. 367871, BD Biosciences, New Jersey, USA) for isolation of peripheral venous blood mononuclear cells (PBMCs) and without anticoagulant (T0, T10, and T30; one tube with 4 mL per sampling time; cat. no. 367812, BD Biosciences, New Jersey, USA) to obtain blood serum. Blood serum was obtained by centrifugation for 10 min at 2,500 x g at room temperature.

### Serum Concentrations of NEFA, BHB, and Haptoglobin

The serum concentrations of BHB and NEFA were analyzed with an automatic analyser (Randox Rx Daytona Chemistry Analyser™, Crumlin, UK) using Randox® commercial kits (Randox Laboratories, Crumlin, UK) for BHB (Randox, cat. n. RB 1007) and NEFA (Randox, cat. n. FA115). The cutoff value established to determine ketosis was 1.2 mmol/L for BHB and 0.8 mmol/L for NEFA, as previously described by Roberts et al. (17).

Furthermore, the serum concentrations of Hp were measured by means of a colorimetric procedure that measures the formation of haptoglobin–hemoglobin complexes and estimates the differences in peroxidase activity, as previously described (18).

### Endometrial Cytology

To assess uterine health, endometrial cytology (EC) was performed with the aid of the Cytobrush® cervical brush (Kolplast, Itupeva, Brazil). Collection of uterine contents was performed as described by Martins et al. (19), which consisted of collecting samples using a human gynecological brush coupled to a universal semen applicator with a stainless steel rod, with protection by a French sheath and sanitary shirt. Then, after passing through the cervix and reaching the base of the uterine body, the sanitary liner was broken, and the applicator plunger was pushed forwards, exposing the brush, which was in contact with the endometrium, was rotated clockwise, and then removed as described by Moura et al. (20). Subsequently, smears with light circular movements were performed on slides with the uterine content obtained with the Cytobrush® cervical brush (Kolplast, Itupeva, Brazil), and subsequently, the prepared slides were stained with panoptic fast stain for evaluation of cellularity by optical microscopy (Primo Star 1, Carl Zeiss, São Paulo, Brazil). The EC was determined by evaluating the percentage of neutrophils from 100 cells at 100X magnification. For the present study, animals with >18% neutrophils in the uterine cytology of the samples collected at T20 and T30 were considered to have a diagnosis of endometritis, as previously described based on examinations of EC in dairy cows between 20 and 33 days postpartum (21–23).

### Expression of PD-1 and CTLA-4 in the T Lymphocyte Population

Initially, PBMCs were isolated by a Ficoll-Paque™ PLUS density gradient (GE Healthcare, Darmstadt, Germany) according to the manufacturer's recommendations. The PBMCs of each sample were transferred to two 5-mL round-bottom polypropylene tubes (A and B), 12 × 75 mm, suitable for flow cytometry, in which they were incubated for 30 min at room temperature with the primary antibodies. Tube A contained 1 μL of the primary *mouse* anti-*bovine* IgG1 antibody CD3 (clone MM1A, cat. N. BOV 2009, Washington State University Monoclonal Antibody Center, USA) and *goat* anti-*human* PD-1 with crossreaction with cattle (diluted 1/10 in PBS with 1% heat sterile inactivated fetal bovine serum and 0.09% sodium azide; cat. No. LS-C55247-100, LSBIO, USA), and tube B contained 1 μL of the primary *mouse* anti-*bovine* IgG1 CD3 (clone MM1A, cat. no. BOV 2009, Washington State University Monoclonal Antibody Center, USA) and *goat* anti-*human* CTLA-4 crossreactive with cattle (diluted 1/10 in PBS with 1% heat-inactivated sterile-filtered fetal bovine serum and 0.09% sodium azide; cat. n. AF-386-PB, R&D Systems, USA). After the incubation period, the cells were washed with PBS and centrifuged at 250 × g for 8 min at 4 °C, the supernatant was discarded, the cells were resuspended in 100 μL of PBS, and goat anti-mouse IgG1-conjugated secondary antibodies conjugated with PE-Texas Red (cat. No. *M32017*, Thermo Fisher, USA) and *crossadsorbed* donkey IgG conjugated with Alexa Fluor 488 (cat. No. A11055, Thermo Fisher, Carlsbad, USA) were added. The samples were incubated at room temperature for 30 min. After the incubation period, the cells were washed with PBS and centrifuged at 250 × g at 4 °C for 8 min. The supernatant was discarded, and the cells were resuspended in 300 μL of PBS with 1% heat-inactivated fetal bovine serum. The sample readings were performed using a BD FACSCanto™ II flow cytometer (BD Biosciences, New Jersey, USA). For this assay, 10,000 cells from each sample were examined. An unstained control, secondary antibody control, isotype control, and single-stained PBMC samples were also prepared as compensation controls. Flow Jo Tree Star software (FlowJo - Treestar 10.5.3 for Windows, Tree Star Inc., Ashland, OR, USA) was used to analyse the data. An example of our gate strategy for the determination of PD-1 expression in T lymphocytes is shown in Supplementary Figure 1. The relative geometric mean fluorescence intensity (GMFI) was chosen because it was far more discriminatory than the percentage of positive cells. The GMFI accurately measures the brightness of the stained cells and consequently indicates the number of receptors per cell (24).

### Statistical Analysis

Data distribution was initially assessed using the *Shapiro–Wilk* test. The parametric distribution data were subjected to ANOVA followed by the *Student–Newman–Keuls* test to compare each variable analyzed at different time points. The variables with a non-parametric distribution were compared by the *Kruskal–Wallis* test, followed by *Dunn's* test, to verify significance (*P* ≤ 0.05), except when indicated. The correlations between the variables analyzed were examined by means of the Pearson and Spearman correlation tests for parametric and non-parametric data, respectively. The statistical software GraphPad Prism 9.0 was used (GraphPad software, Inc., San Diego, USA).

To investigate the association between the expression of CLTA-4 and PD-1 in T lymphocytes, with endometritis indicated as the percentage of neutrophils > 18% in EC at T20 and T30, generalized linear regression was used, with the dependent variable being disease (percentage of neutrophils > 18%) and explanatory variables being the sampling time, parity, and the farm/breed. All of these analyses were performed in the R program [R (25)] and the tidyverse (26) and broom (27) packages. The results are expressed as the mean ± standard error, and α = 5% was used for all analyses, except when indicated.

## Results

### Descriptive Analysis

In the present study, no significant differences were observed in the generalized linear regression model between multiparous and primiparous cows, with no effect of dairy farms/breeds on the variables analyzed. Out of 26 enrolled dairy cows, 16 (61.54%) and six (23.08%) dairy cows developed cytological endometritis at T20 and T30, respectively, i.e., showed a high percentage of neutrophils (>18%) in the endometrial cytology. The diagnosis of ketosis determined by the serum concentrations of BHB and NEFA was detected at T0 in one (3.85%) and seven (26.92%) dairy cows, at T10 in four (15.38%) and three (11.54%) dairy cows, and at T30 in two (7.69%) and two (7.69%) dairy cows, respectively.

### Diagnosis of Ketosis and Its Relationship With the Expression of PD-1 and CTLA-4 by Blood T Lymphocytes

The analyzed time points did not affect the serum concentration of BHB (*P* = 0.45); however, the serum NEFA concentration was higher at T0 than T10 (*P* = 0.02; Supplementary Figure 2). In addition, high concentrations of BHB (≥ 1.2 mmol/L) and NEFA (≥ 0.8 mmol/L) were not associated with CTLA-4 or PD-1 expression in blood T lymphocytes in the generalized linear regression model.

### Diagnosis of Cytological Endometritis and Its Relationship With the Expression of PD-1 and CTLA-4 by Blood T Lymphocytes

**Figure 1:**
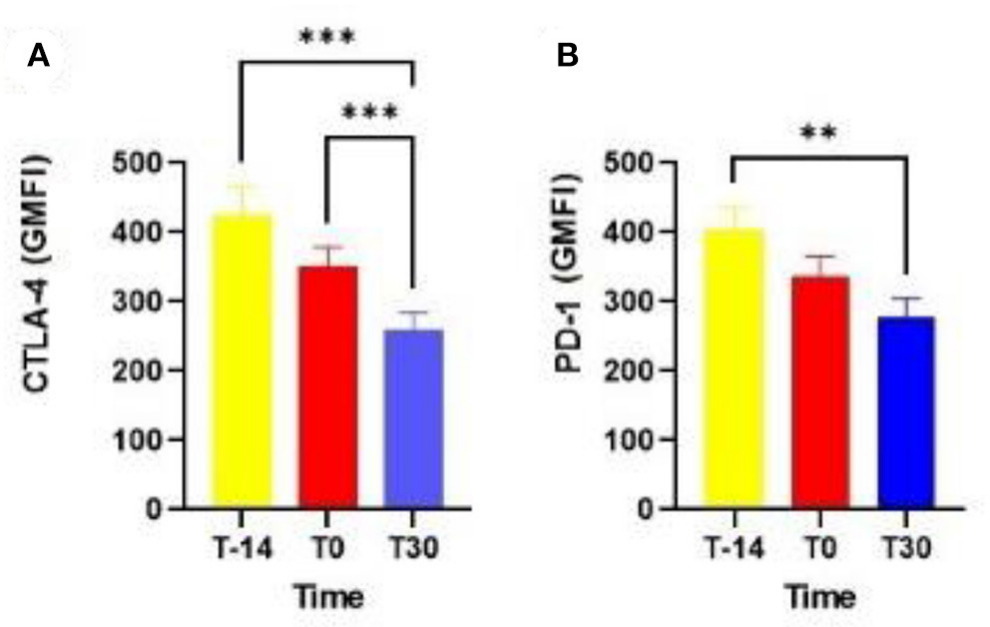
Expression of CTLA-4 **(A)** and PD-1 by blood T lymphocytes in dairy cows at T-14, T0, and T30 **(B)**. CTLA-4: cytotoxic T lymphocyte-associated antigen-4; PD-1, programmed cell death protein 1; T-14 = 14 days before delivery; T0 = day of delivery; T30 = 30 days postpartum; GMFI, geometric mean fluorescence intensity. **P < 0.01; ***P < 0.0001.

One notable result was the higher expression of CTLA-4 in T lymphocytes at T-14 (*P* = 0.0007) and T0 (*P* = 0.0004) than at T30 (Figure 1A). The expression of PD-1 in T lymphocytes at T-14 was also higher than that at T30 (*P* = 0.003; Figure 1B). The percentage of neutrophils in the EC at T10 (27.50 ± 5.90), T20 (30.31 ± 5.26), and T30 (14.65 ± 4.00) also did not differ among the analyzed time points (*P* = 0.12).

**Figure 2:**
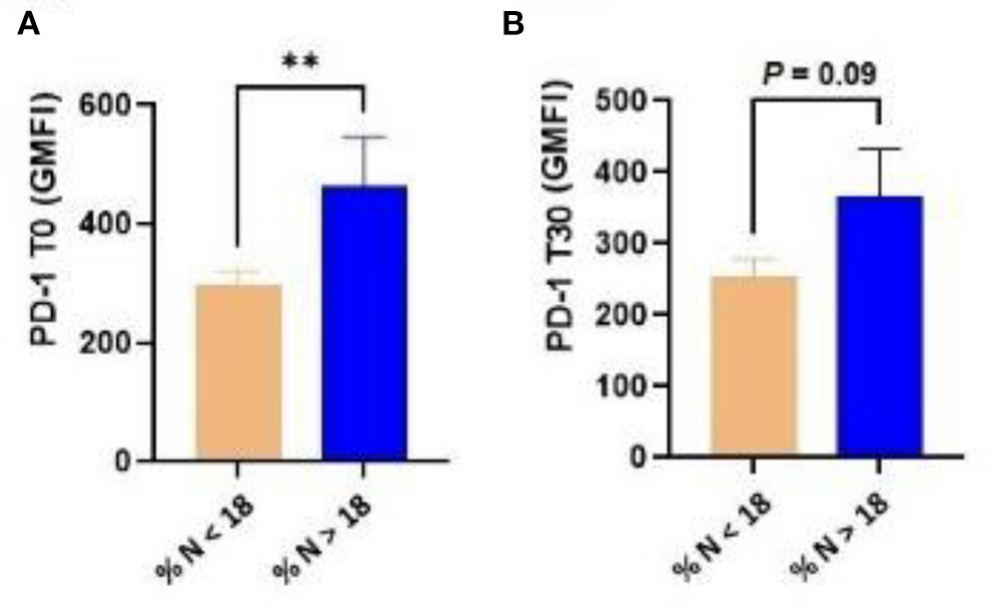
Dairy cows diagnosed with cytological endometritis [i.e., percentage of neutrophils higher than 18 (% N > 18)] at T30 had a higher expression of PD-1 in blood T lymphocytes at both T0 **(A)** and T30 **(B)**. PD-1, programmed cell death protein 1; T0 = day of delivery; T30 = 30 days postpartum; GMFI, geometric mean fluorescence intensity. **P < 0.01.

In the generalized linear regression model, the diagnosis of cytological endometritis at T20 showed an association with the expression of PD-1 in T lymphocytes (*P* = 0.02) and a trend with the expression of CTLA-4 in T lymphocytes (*P* = 0.086) at parturition. In addition, dairy cows diagnosed with cytological endometritis (neutrophil percentage > 18 determined by EC) at T30 showed a higher expression of PD-1 by T lymphocytes at T0 (*P* = 0.009) as well as a tendency for a higher expression of PD-1 at T30 (*P* = 0.09; Figure 2).

The expression of CTLA-4 in blood T lymphocytes at T-14 was correlated with the percentage of neutrophils at T30 (r = 0.56; *P* = 0.01). Furthermore, at T0, the expression of CTLA-4 in T lymphocytes was again correlated with the percentage of neutrophils T30 (r = 0.50; *P* = 0.03). Similarly, at T0, the expression of PD-1 in T lymphocytes showed a significant correlation with the percentage of neutrophils at T30 (r = 0.62; *P* = 0.006).

### Serum Concentration of Haptoglobin and Its Relationship With the Expression of PD-1 and CTLA-4 by Blood T Lymphocytes

The analyzed time points did not affect the serum concentration of haptoglobin (*P* = 0.38). The expression of CTLA-4 in blood T lymphocytes at T-14 was correlated with the serum concentration of haptoglobin at T30 (r = 0.68; *P* = 0.002). Furthermore, at T0, the expression of CTLA-4 in T lymphocytes was again correlated with the serum concentration of haptoglobin T30 (r = 0.66; *P* = 0.003). Additionally, at T-14, the expression of PD-1 in T lymphocytes was correlated with the serum concentration of T30 haptoglobin (r = 0.62; *P* = 0.006). Similarly, at T0, the expression of PD-1 in T lymphocytes showed a significant correlation with the serum concentration of haptoglobin T30 (r = 0.72; *P* = 0.0007).

In addition, the serum concentration of haptoglobin at T10 was correlated with the expression of CTLA-4 in T lymphocytes at T30 (r = 0.74; *P* = 0.0005) and the expression of PD-1 in T lymphocytes at T30 (r = 0.63; *P* = 0.005). The serum concentration of haptoglobin at T30 was correlated with CTLA-4 expression in T lymphocytes at T30 (r = 0.65; *P* = 0.41) and PD-1 expression in T lymphocytes at T30 (r = 0, 65; *P* = 0.004). The predictive values of haptoglobin at distinct time points from diagnosed endometrial cytology are shown in Supplementary Table 1.

## Discussion

The postpartum period in dairy cows represents the successful completion of a long gestation period in which there is a challenge to uterine immunity. For instance, during pregnancy, host immunity must be suppressed to limit the immune response against the halogen concept, in which the differentiation of regulatory T cells is crucial to inhibit T-cell responses and the inflammatory process. On the other hand, the immune response must act against infections (28, 29). In this scenario, the immune checkpoints CTLA-4 and PD-1 are widely recognized as negative regulators of T-cell immunity (30). Thus, the higher expression of CTLA-4 and PD-1 in T lymphocytes during the prepartum period in dairy cows in the present study indicates its important role in the maintenance of maternal immune tolerance that occurs during pregnancy, as previously reported in humans (31–34) and rats (35). In fact, T cells play a key role in the induction and maintenance of maternal–fetal tolerance in dairy cows (26) because they are able to differentiate into distinct subsets, including Th1, Th2, Th17, and Treg cells, and during normal pregnancy, there is a dominance of Th2 and Treg-type cytokines, considered the main mechanism of induction of tolerance of the fetus; however, in spontaneous abortion, Th1-type cytokines are dominant (36, 37). At this point, progesterone, although not measured in the experiment, seems to play an important role in the maternal immune tolerance process in dairy cows (38). Although no study has investigated the link between progesterone and the expression of immune checkpoints in T cells in dairy cows, the induction of the expression of CTLA-4 and PD-1 immune checkpoints in T lymphocytes by progesterone has been previously demonstrated in mice (39).

In addition, it is clear that both the intensity of uterine immunosuppression that occurs during the prepartum period and the rapid restoration of defense mechanisms determine whether the animal will develop metritis or endometritis in the postpartum period (38). This may explain, at least in part, the relationship between the expression of CTLA-4 and PD-1 immune checkpoints in T lymphocytes in the prepartum and at partum with endometrial cytology at T20 and T30, an important tool used in the diagnosis of bovine endometritis. Thus, we should highlight that our study showed that the levels of PD-1 and CTLA-4 expression in T cells at parturition could predict the occurrence of cytological endometritis in dairy cows, paving the way for the early identification and diagnosis of susceptible cows. Furthermore, the increased expression of CTLA-4 and PD-1 immune checkpoint markers in T lymphocytes may explain the susceptibility of dairy cattle to infection in the prepartum and calving periods, especially when considering the importance of T cells for mucosal immunity (40). Unsurprisingly, the prevalence of new intramammary infections in the prepartum period and at partum is also very high (41) and may be explained at least in part by the findings of the present study; this possibility deserves further research. Additionally, the aforementioned finding is supported by the correlation between the expression of the *immune* checkpoint markers CTLA-4 and PD-1 in T lymphocytes during the prepartum period and with the increase in the serum concentration of Hp, which is the most important acute-phase protein in cattle (14, 42) and is usually associated with inflammatory processes resulting from infections.

The negative energy balance gains prominence in the postpartum period, leading to the occurrence of metabolic disorders and infectious diseases such as hypocalcaemia, abomasal displacement, mastitis, and metritis (43, 44) due at least in part to negative implications for the immune response (28, 45). However, the present study did not find a relationship between the occurrence of ketosis and the expression of CTLA-4 and PD-1 in blood T lymphocytes, suggesting that the recognized immunosuppression related to the negative energy balance is not related to the expression of the immune checkpoints studied here in T lymphocytes. Therefore, considering the limited number of dairy cows diagnosed with ketosis in our study, we should be careful when interpreting our findings.

We should also note that the inhibition of T lymphocytes by these immune checkpoints is reversible, for example, by the use of anti-programmed death-ligand 1 (PD-L1), anti-PD-1, or CTLA-4 monoclonal antibodies, opening new frontiers for the treatment and prevention of diseases. Thus, the functional inhibition of immunosuppressive factors represents a viable tool to reactivate immune cells in a state of tolerance, providing a new therapeutic strategy for chronic infections and tumor diseases or as an approach to increase the efficacy of vaccines (46).

## Conclusion

The present study pointed to the important role of the expression of PD-1 and CTLA-4 in T lymphocytes during the periparturient period and its effect on uterine health. Thus, our findings suggest that immune checkpoints play a key role in immunological tolerance during bovine pregnancy, which in turn, if they are highly expressed in T lymphocytes, may inhibit the immune response to such an extent that they lead to negative implications for uterine health. Thus, the present study represents an initial step that paves the way for the development of new therapeutic alternatives directed by the host with the objective of increasing the resistance of dairy cattle to infections in this critical period of their life.

## Data Availability Statement

The original contributions presented in the study are included in the article/Supplementary Material, further inquiries can be directed to the corresponding author/s.

## Ethics Statement

The animal study was reviewed and approved by Universidade Federal da Paraíba. Written informed consent was obtained from the owners for the participation of their animals in this study.

## Author Contributions

CS performed all the experiments and wrote and edited the manuscript. EL, RO, BO, RR, and MM performed the experiments. DL performed all statistical analyses. MB, ER, IM, FS, and AF designed and supervised the studies and drafted and edited the manuscript. All authors have read and agreed to the published version of this manuscript.

## Funding

This work was financed by Public Call n. 01/2021 Produtividade em Pesquisa PROPESQ/PRPG/UFPB, project code: 13303-2020.

## Conflict of Interest

The authors declare that the research was conducted in the absence of any commercial or financial relationships that could be construed as a potential conflict of interest.

## Publisher's Note

All claims expressed in this article are solely those of the authors and do not necessarily represent those of their affiliated organizations, or those of the publisher, the editors and the reviewers. Any product that may be evaluated in this article, or claim that may be made by its manufacturer, is not guaranteed or endorsed by the publisher.
