# Supplementary material for: Brief Research Report: Expression of PD-1 and CTLA-4 in T Lymphocytes and Their Relationship With the Periparturient Period and the Endometrial Cytology of Dairy Cows During the Postpartum Period
Source: Front Vet Sci. 2022 Jul 22;9:928521. doi: 10.3389/fvets.2022.928521 (PMC9353034; doi:10.3389/fvets.2022.928521)
Supplement: Supplementary file 1 [file Data_Sheet_1.docx]

**
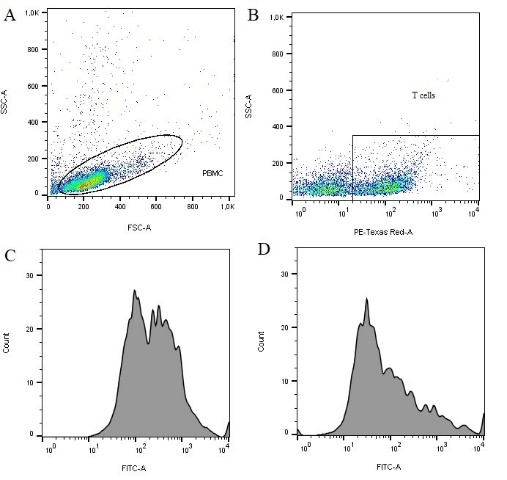
**

**Supplementary Figure 1.** A sequential gating approach was used to measure the expression of programmed cell death protein 1 (PD-1) in T cells. First, peripheral blood mononuclear cells were identified after excluding most of the debris **(A)**. Subsequently, T lymphocytes were identified using a two-step fluorescent immunolabelling protocol that included a primary anti-bovine monoclonal antibody (Ab) specific for T lymphocyte identification (CD3) and a secondary antibody coupled to a long-wavelength fluorescent probe [PE (phycoerythrin)-Texas Red] **(B)**. The histograms depict the expression of PD-1 in blood T cells 14 days before **(C)** and 30 days after **(D)** parturition, as determined by a two-step fluorescent immunolabelling protocol utilizing a primary antibody specific for PD-1 that crossreacts with bovine cells and a secondary antibody coupled to a long-wavelength fluorescent probe (Alexa Fluor 488).

**
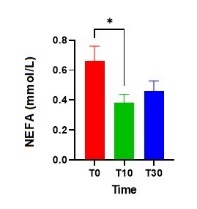
**

**Supplementary Figure 2.** Serum concentration of nonesterified fatty acids (NEFA) at 10 (T10), 20 (T20), and 30 (T30) days postpartum.

Supplementary Table 1. Predictive values of haptoglobin (Hp) at distinct time points to diagnose cytological endometritis at 20 (T20) and 30 (T30) days postpartum in dairy cows.

| Time points of the diagnosis of cytological endometritis | Time points of Hp measurements | Cutoff  (mg/dL) | Area under the ROC curve (mean + SEM) | Sensitivity (%) | Specificity (%) |
| --- | --- | --- | --- | --- | --- |
| T20 | T0 | 1.59 | 0.68 + 0.11 | 76.47 | 66.67 |
|  | T10 | 0.96 | 0.62 + 0.13 | 88.24 | 55.56 |
|  | T30 | 0.985 | 0.63 + 0.12 | 75.00 | 70.00 |
| T30 | T0 | 1.295 | 0.57 + 0.12 | 66.67 | 55.06 |
|  | T10 | 1.305 | 061 + 0.12 | 66.67 | 60.00 |
|  | T30 | 1.45 | 0.59 + 0.13 | 66.67 | 50.00 |

ROC = receiver operating characteristics; Cutoff: cutoff value of haptoglobin that maximized the sensitivity and specificity; T0: at parturition; T10: 10 days after parturition.
